# Pupil adaptation corresponds to quantitative measures of autism traits in children

**DOI:** 10.1038/s41598-017-06829-1

**Published:** 2017-07-25

**Authors:** Antoinette Sabatino DiCriscio, Vanessa Troiani

**Affiliations:** Geisinger Health System, Geisinger Autism and Developmental Medicine Institute (ADMI), Lewisburg, PA USA

## Abstract

The pupil is known to reflect a range of psychological and physiological variables, including cognitive effort, arousal, attention, and even learning. Within autism spectrum disorder (ASD), some work has used pupil physiology to successfully classify patients with or without autism. As we have come to understand the heterogeneity of ASD and other neurodevelopmental disorders, the relationship between quantitative traits and physiological markers has become increasingly more important, as this may lead us closer to the underlying biological basis for atypical responses and behaviors. We implemented a novel paradigm designed to capture patterns of pupil adaptation during sustained periods of dark and light conditions in a pediatric sample that varied in intellectual ability and clinical features. We also investigate the relationship between pupil metrics derived from this novel task and quantitative behavioral traits associated with the autism phenotype. We show that pupil metrics of constriction and dilation are distinct from baseline metrics. Pupil dilation metrics correlate with individual differences measured by the Social Responsiveness Scale (SRS), a quantitative measure of autism traits. These results suggest that using a novel, yet simple, paradigm can result in meaningful pupil metrics that correlate with individual differences in autism traits, as measured by the SRS.

## Introduction

Pupil response has a longstanding history of being used as a peripheral indicator of underlying neurologic and physiologic function. In response to changes in light, the pupil reflexively constricts or dilates, thus controlling the amount of luminance falling upon the retina^1, 2^. Pupil diameter is influenced by stimulus-driven or environmental factors, such as ambient light, motion, color, and contrast^3–5^. The amplitude and velocity of pupil changes are influenced by the intensity of the stimulus employed^6^ as well as an individual’s age^7–9^. Changes in pupil response can also reflect higher-level cognitive abilities, such as attention or the exertion of effort^10–13^, and the influence of several distinct cognitive variables can be captured within a single paradigm^14^. Furthermore, the pupil is thought to reflect changes in neurotransmitter release, particularly norepinephrine^15–17^. Thus, seemingly simple pupil changes can be seen as a “window to the brain” and can serve as a non-invasive measure of cognition and potentially, a proxy for the neural response in certain brain regions.

Several previous studies have investigated atypical pupillary response in autism using eye tracking technology and come upon a diverse pattern of results. Rubin^18^ was one of the earliest to assess pupil size during dark and light adaptation in children and reported that children with autism had *smaller* pupil sizes during dark adaptation and slower constriction during light adaptation. In contrast, several studies have demonstrated a difference in *baseline pupil diameter* (i.e. pupil size in the absence of a task or stimulus) relative to age-matched controls^19, 20^. Other groups have found no difference in baseline pupil diameter between children with and without autism^21^. In addition to examining differences in baseline pupil response, studies have examined task-induced differences in pupil response and noted atypical pupil responses in autism^21–30^. Discrepancies across reported results described above may be attributed to differences in task parameters and stimulus properties known to influence both baseline and task-induced pupil response, such as color and contrast^3–5^.

Other work has examined the pupillary light reflex (PLR), specifically. The PLR is an automatic sensory response that allows the eye to adjust the amount of light that reaches the retina. The classic paradigm involves measuring pupil response following a very brief (120 ms) flash of light^31, 32^, but other stimulus formats varying in contrast, luminance, and spatial frequency have also been used^33^. An atypical PLR has been linked to ASD and can even be used to discriminate ASD from controls with high accuracy^25^. However, atypical pupil responses are also found in children with other neurodevelopmental disorders^34^. Thus, the ability to discriminate between autism and controls using pupil responses may be driven by other characteristics that differ between the groups, such as differences in intellectual ability.

Despite current knowledge regarding atypical pupil response in ASD, little is known regarding the individual differences in pupil response as it relates to core diagnostic features of ASD. The disorder is based upon observed deficits in social interaction and communication in conjunction with the presence of restricted interests and repetitive behaviors. However, a varied range of aberrant behaviors (i.e. deficits in arousal, cognition, and disproportionate strengths in visual perception) have also become central to the description of autism. ASD features have also been described in individuals who do meet criteria for a diagnosis. Autism traits that are below the clinical threshold for diagnosis and commonly found in at least one parent of children with autism were first described as the broader autism phenotype (BAP). More recently, this term has been adopted to describe subclinical traits in the broader population that are continuously distributed^35–37^. This diverse yet continuous spectrum of neurobehavioral traits may be coupled with a diverse range of underlying neuronal features.

**Figure 1:**
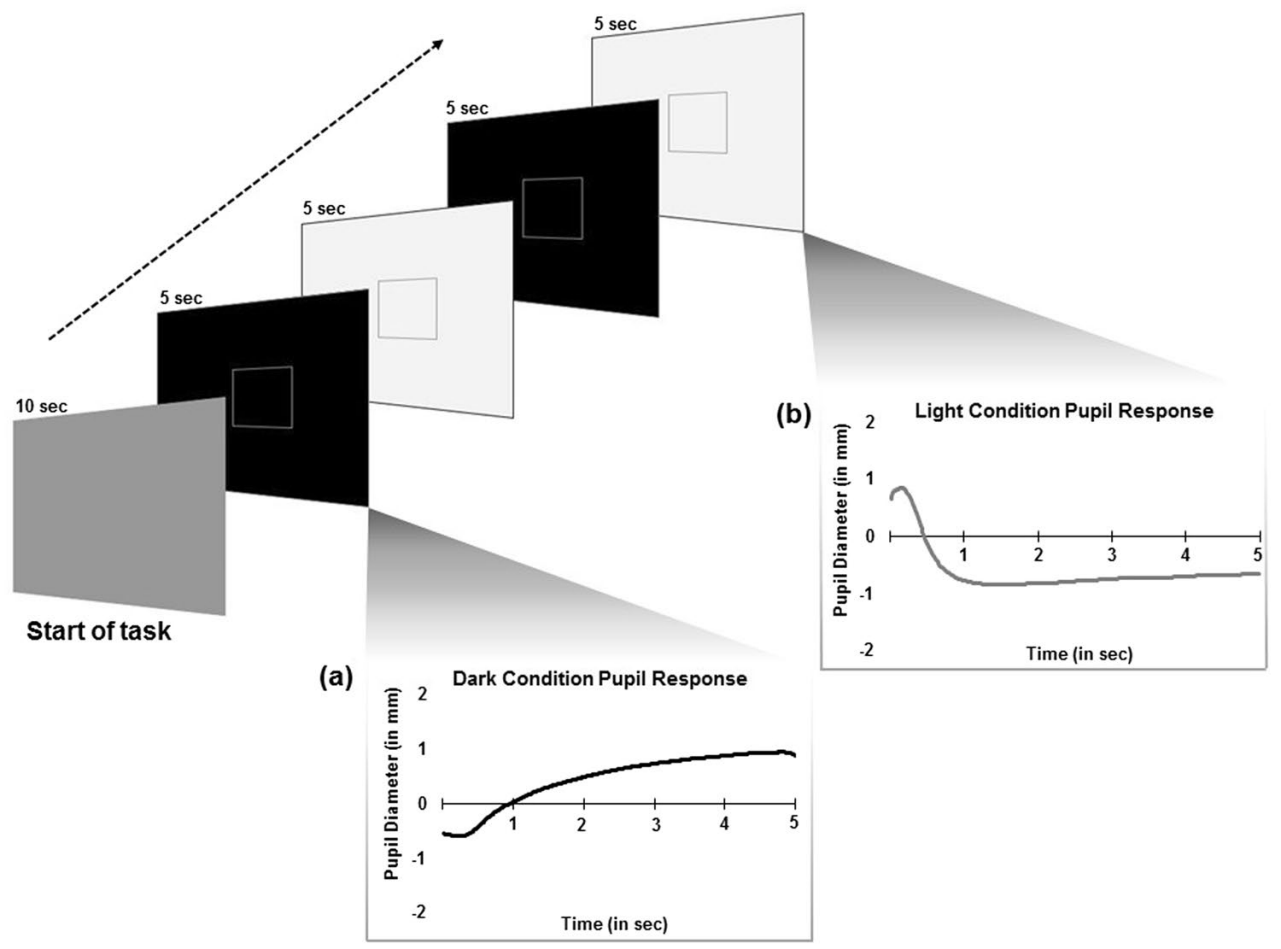
Task schematic including sample stimuli. The eye tracking task began with a 10 second gray screen from which baseline pupil diameter was extracted. Pupil response was measured for (**a**) “dark condition” stimuli and (**b**) “light condition” stimuli.

In order to better understand whether pupil changes are related specifically to quantitative measures of autism traits, we designed an eye tracking task that measured pupillary changes in response to a sustained stimulus presentation (alternating black and white screens, see Fig. 1). Additionally, we sought to capture meaningful individual differences in pupil adaptation across dark and light conditions and investigate the relationship with quantitative behavioral traits associated with the autism phenotype as measured by the Social Responsiveness Scales-2^nd^ Edition (SRS-2)^38, 39^. We predicted that reflexive and spontaneous changes in pupil constriction and dilation would be associated with autism traits.

## Results

**Figure 2:**
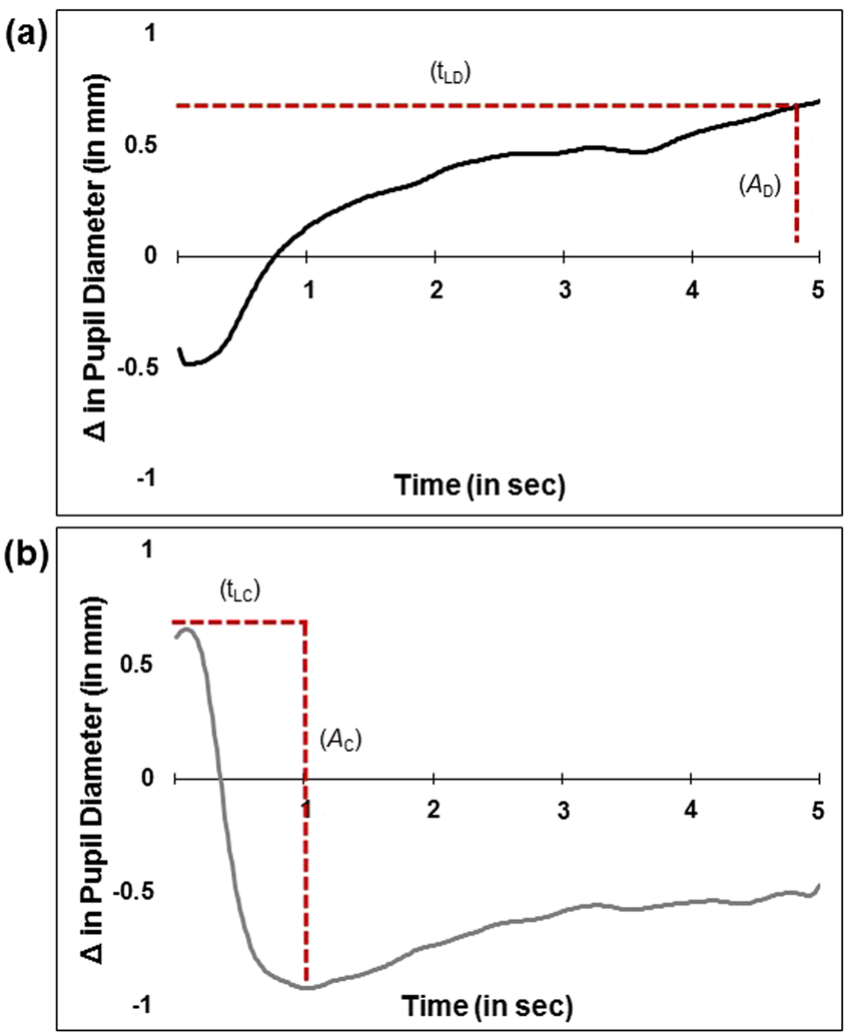
An illustration of the pupillary data from a single participant and pupil metrics (t_L_ and *A*) that were extracted individually for each participant across both conditions. (**a**) Latency to reach maximum dilation (t_DL_) during the dark condition and amplitude of dilation (*A*_D_) during the dark condition. (**b**) Latency to reach maximum constriction (t_CL_) during the light condition and amplitude of constriction (*A*_C_) during the light condition.

Two components of the pupil adaptation response were of interest in the current analysis: (1) the *amplitude* of dilation (*A*_D_) or constriction (*A*_C_) across each condition as well as (2) the *latency* to reach maximum dilation (t_DL_) or constriction (t_CL_) across each condition (See Fig. 2 for visual depiction of these metrics). These metrics were derived from work on the PLR, but it is important to note that our latency metric should not to be considered synonymous with the PLR, given our use of sustained rather than transient stimulus presentation. Each of these pupil metrics was compared across conditions in a multivariate analysis of variance (MANOVA) in order to confirm the effect of our light and dark stimulus conditions on producing appropriate physiological responses (i.e. constriction upon exposure to the light condition and dilation during exposure to the dark condition). We go on to identify whether individual differences in pupil metrics predict quantitative measures of autism traits assessed via the Social Responsiveness Scale-2^nd^ Edition (SRS-2)^38, 39^.

### Differences in pupil metrics between Light and Dark Conditions

We examined whether stimulus conditions (dark and light conditions) elicited appropriate and distinct physiological pupil responses. Given previous reports of gender differences in the lateralization of pupil constriction between males and females^40^, we also explored whether there was an effect of gender on pupil metrics in the current experiment. Results of a 2 (condition) x 2(gender) x 4 (constriction, dilation, constriction latency, dilation latency) multivariate analysis of variance (MANOVA) indicated a main effect of condition (F(4, 77) = 142.87, *p* < 0.001); however, no main effect of gender (F(4, 77) = 0.39, *p* = 0.82, NS) nor a condition x gender interaction (F(4, 77) = 1.56, *p* = 0.19, NS). Pairwise comparisons using a Bonferroni correction for multiple comparisons indicated that the amplitude of constriction was much greater during the light condition as compared to the dark condition, *p* < 0.001, and amplitude of dilation was much greater during the dark condition as compared to the light condition, *p* < 0.001. Latency of maximum constriction in the dark condition was significantly faster than the light condition, *p* < 0.001, which is as expected since participants began dark condition trials at a smaller pupil diameter and responded with a reflexive dilation. However, there was no significant difference in the latency to dilate between dark and light conditions, *p* = 0.88, NS. Thus, these analyses confirmed that our task parameters were appropriate and stimulus conditions resulted in distinct physiological differences in pupil response.

### Individual differences in pupil metrics as predictors of autism traits

We next assessed the collinearity among our pupil metrics and other participant variables (i.e. age IQ, and quantitative measures of autism traits). Results from bivariate correlations are outlined in Table 1. It is important to note that several relationships among our pupil metrics were observed. SRS Total T-score was correlated with the amplitude of constriction (*A*_C_) (r = −0.40, *p* = 0.008), amplitude of dilation (*A*_D_) (r = −0.55, *p < *0.001) and latency of constriction (t_CL_) (r = −0.38, *p* = 0.01). Age was not correlated with FSIQ or pupil metrics with the exception of baseline pupil diameter (r = −0.30, *p* = 0.05). FSIQ was correlated with dilation amplitude (*A*_D_) (r = 0.31, *p* = 0.05) as well as SRS Total T-score (r = −0.55, *p* < 0.001). See Table 1 for complete results.

**Table 1:** Correlation matrix including pupil metrics, age (in years), FSIQ, and SRS Total T-score.

	R value (*p* value)							
	Age	FSIQ	Baseline	t_CL_	t_DL_	*A*_C_	*A*_D_	SRS Total
Age	1.00—							
FSIQ	0.14 (0.39)	1.00—						
Baseline	−0.30* (0.05)	0.07 (0.68)	1.00—					
t_CL_	−0.25 (0.12)	0.20 (0.22)	−0.16 (0.33)	1.00 —				
t_DL_	0.30* (0.05)	0.02 (0.88)	−0.54** (<0.001)	0.08 (0.63)	1.00—			
*A*_C_	−0.02 (0.90)	0.12 (0.46)	−0.42* (0.005)	0.20 (0.20)	0.63** (<0.001)	1.00—		
*A*_D_	−0.04 (0.79)	0.31* (0.05)	−0.13 (0.41)	0.38** (0.01)	0.31* (0.04)	0.66** (<0.001)	1.00—	
SRS Total	0.22 (0.16)	−0.55** (<0.001)	0.08 (0.62)	−0.38** (0.01)	−0.19 (0.23)	−0.40** (0.008)	−0.55** (<0.001)	1.00—

Note: *Indicates correlation is significant at the 0.05 level (two-tailed); **indicates correlation is significant at the 0.01 level (two-tailed).

Abbreviations: Baseline = baseline pupil diameter; t_LC_ = latency to constrict; t_LD_ = latency to dilate; *A*_C_ = amplitude of constriction; *A*_D_ = amplitude of dilation.

Given the degree of correlation between several of these metrics, a stepwise linear regression was run to predict SRS Total T-score from baseline pupil diameter, constriction amplitude (*A*_C_), dilation amplitude (*A*_D_), latency of constriction (t_CL_), latency of dilation (t_DL_), chronological age, and full-scale IQ (FSIQ). A model including both FSIQ and amplitude of dilation (*A*_D_) significantly predicted SRS Total T-score (F(2, 37) = 14.10, *p* < 0.001, *R* ^2^ = 0.43). All other variables were not significant predictors of SRS score (*p*’s > 0.17, NS). See Table 2 for results. Thus, autism symptoms can be predicted by reflexive changes in pupil dilation during dark adaption above and beyond the predictive value of FSIQ.

**Table 2:** Stepwise Linear Regression Analysis to predict SRS Total T-scores.

	β	R^2^	Adj R^2^	CI	p-value
FSIQ	−0.37	0.30	0.29	−0.59−0.14	0.002
Dilation (A_D_)	−11.60	0.43	0.40	−19.74–3.46	0.006

*β* = Beta weight; *R* ^*2*^ = explained variance; *Adj R* ^*2*^ = adjusted variance; *CI* = 95% confidence internal.

### Relationship between amplitude of pupil adaptation and SRS-2 scores

**Figure 3:**
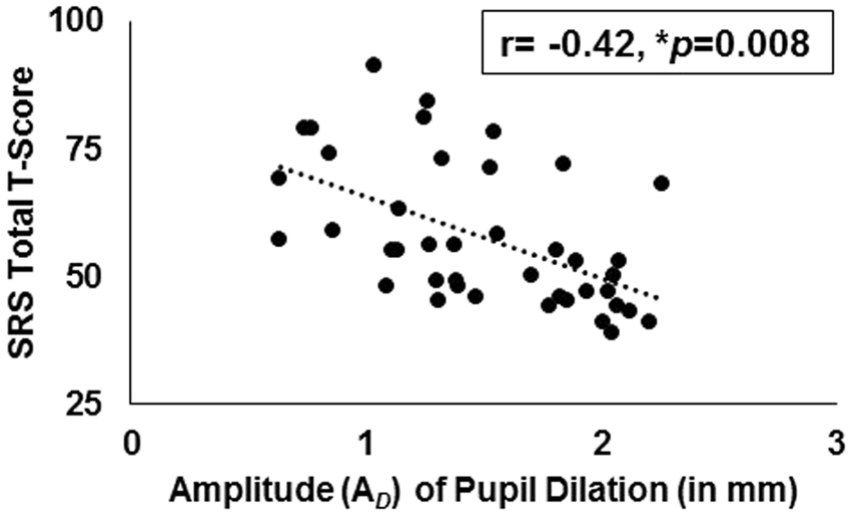
Amplitude Results. Scatterplot indicating the relationship between the *amplitude* of pupil *dilation* (*A*_D_) and SRS Total t-score (statistics reported are a result of a partial correlation controlling for age and FSIQ).

Given results from the stepwise linear regression, we examined the relationship between *amplitude* of pupil dilation during the dark condition and SRS-2 total and subscale scores via partial correlations, controlling for age and IQ. Amplitude of dilation of the pupil in the light condition was found to be significantly related to SRS Total T-score (r = −0.42, *p* = 0.008, see Fig. 3) as well as all SRS subscales (SCI: r = −0.46, *p* = 0.003; Social Awareness: r = −0.34, *p* = 0.04: Social Cognition: r = −0.51, *p* < 0.001; Social Communication: r = −0.47, *p* = 0.003) with the exception of Social Motivation (r = −0.30, *p* = 0.07, NS) and RBRI (r = −0.25, *p* = 0.12, NS). Additional results from a partial correlation, controlling for age and IQ, among all pupil metrics and SRS scores can be found in the supplement.

### ASD subgroup analysis

While the focus of this study was to assess quantitative traits of autism and individual differences in pupil adaptation, we also confirmed that our main effect differentiated an ASD subsample (N = 12) from those participants not diagnosed with an ASD (N = 30). Individuals carrying an ASD diagnosis also had significantly reduced dilation amplitude relative to the non-ASD group (t(40) = 3.66, *p* = 0.001). This effect is consistent with previous work that finds pupil differences based on dichotomous groups of patients with and without ASD.

## Discussion

In the current study, we find a relationship between measures of pupil adaptation in light and dark stimulus conditions and quantitative measures of autism traits within a pediatric sample. While atypical pupil response has been studied previously within autism across a variety of contexts, we made use of a simplified passive viewing paradigm in order to quantify reflexive changes in pupil constriction and dilation evoked by alternating light and dark stimuli. We show inverse relationships between autism traits measured via the SRS and amplitude of pupil dilation, demonstrating that individual differences in pupil adaptation scale with quantitative autism traits that extend beyond traditional and categorical diagnoses for autism. Common practice across studies of pupil response in autism is to focus on the relative comparison of individuals with ASD to a non-clinical control population^19, 20, 25^. While this practice has proven effective in capturing atypical pupil response in ASD, dichotomizing groups in such a way fails to capture meaningful individual differences in the relationship between pupil response and clinically relevant behavioral phenotypes. Thus, we chose to investigate this phenomenon in a sample with a range of abilities, including both a clinically- and community- ascertained sample.

In this study, we aimed to assess patterns of pupil constriction and dilation using alternating and sustained periods of dark and light conditions (i.e. white and black screens). The sustained nature of our stimulus presentation is a key difference between PLR paradigms and our experiment. While PLR paradigms have been used to show differences between groups of children with and without ASD^18, 29^, to our knowledge, no study has linked PLR to quantitative measures of ASD. The sustained presentation may be the factor that allowed us to capture a relationship between individual differences in autism traits and metrics of pupil adaptation. The amplitude of pupil dilation in the current paradigm was found to be a significant predictor of autism features. Significant correlations were found for dilation amplitude and the SRS Total Score, as well as most SRS subscores. Additional correlations between our other pupil metrics and SRS scores can be found in the supplement. Interestingly, the RBRI subscale was unrelated to any of the pupil metrics using this passive viewing task. This result may appear counter to the suggestion that the RBRI domain is linked with atypical pupil responses that reflect hyperfocus and circumscribed interests^23^. However, it is important to emphasize that it is plausible that certain physiological pupil responses within task-based paradigms are linked to one domain of autism traits, while others are associated with baseline pupil diameter and/or automatic, reflexive responses. Future work should test the same individuals on multiple experiments in order to tease apart whether pupil responses evoked from different types of tasks are linked with specific symptom domains of the autism phenotype.

Findings of atypical pupil diameter in ASD have been attributed to discordant autonomic function^25, 41^. However, the degree to which differences in baseline or “resting” pupil diameter is characteristic of autism remains uncertain, as findings are inconsistent across studies^20, 25^. For example, Fan *et al*.^25^, reported no significant difference in initial pupil diameter between ASD and control groups. These results stand in contrast to a study by Martineau *et al*.^20^, showing significantly smaller baseline pupil size in the ASD group relative to matched controls. Differences in baseline pupil size are also thought to reflect aberrant function of subcortical or lower-order systems (i.e. the locus coeruleus and noradrenergic pathways) known to play a role in pupillary changes^19^. It should be noted that the aforementioned studies use a small N (10 or less children with ASD)^20, 25^ or extract baseline pupil size from a task using stimuli that are known to evoke differences within the ASD population (i.e. faces)^19, 20^. Thus, although we do not find that baseline pupil diameter is linked with autism traits in the current study, we believe this adds to the growing body of evidence that indicates atypical pupil responses in ASD.

One fundamental question is how underlying neuronal features assessed via pupil response are related to autism traits, specifically those associated within the social domain assessed via the SRS-2. While research on the biological underpinnings of autism have primarily focused on social and motivational circuitry (medial frontal cortex; amygdala and other limbic structures; fusiform)^42–45^, other theoretical explanations highlight the role of the autonomic nervous system in generating hyper aroused states that may contribute to deficits in social cognition and social impairment^46, 47^. That is, although atypical autonomic function may not seem related to social functioning, it is thought that appropriate autonomic function and the ability to recognize changes in our own internal states may be a necessary biological precursor to intact emotion recognition. This is supported by work outside of autism in which atypical patterns of arousal have been observed in conjunction with impairments in social-problem solving skills^48^. Thus, discordant arousal states that emerge from aberrant function of the parasympathetic and sympathetic nervous systems may be tied to maladaptive social features central to the autism phenotype.

There are several caveats in the current investigation that should be addressed in future studies. Despite the fact that reported results accounted for age, gender, and IQ, our current sample includes a relatively wide developmental age range and a wide range of IQs. Research has acknowledged the effect of age on pupillary changes in adults^6, 9^; however, additional research must be done to characterize critical changes in pupil response across typical and atypical development within younger populations. Along the same lines, additional peripheral indicators of functional state (i.e. skin conductance, baseline cardiac activity) should be collected in order to account for individual differences across other variables pertaining to autonomic function and arousal state that may be influencing reflexive changes in pupil diameter.

To our knowledge, this is the first time a pupil adaptation task of this kind was implemented to capture individual differences and quantitative traits within a pediatric sample with variable levels of functioning. A majority of the current research in ASD and other developmental disorders focuses largely on higher functioning individuals. Typically, this is defined as individuals with an IQ ≥ 80 while those with an IQ < 80 are considered to be lower functioning ^49, 50^. Our current sample includes a rather wide range of IQs (ranging from 55 to ~130) resulting in a rather high group average FSIQ. However, in contrast to previous work on ASD and the conventional definitions of high versus low functioning, our sample includes individuals with variable levels of functioning and represents a wide range of ASD features. This work provides a valuable first step regarding the use of a simplified, passive eyetracking task such as this for research within a clinical population. In order to continue to quantify meaningful individual differences in pupil response and their relationship to the clinical features associated with ASD, future research with such simplified paradigms is needed in larger samples with an even broader range of language and intellectual abilities.

Pupil metrics can be used to assess meaningful individual differences across clinically relevant behavioral phenotypes. One key difference between our paradigm and studies of the pupillary light reflex is the sustained nature of our stimulus presentation. This sustained presentation may contribute to capturing meaningful individual differences in pupillary physiology that is relevant to quantitative traits associated with the autism phenotype. Overall, this work adds to the growing body of evidence that links the dimensional measurement of quantitative traits associated with autism and atypical visual sensory phenomena^51, 52^.

## Methods

### Participants

A total of 49 children were recruited for the study. We were unable to obtain eye tracking data in 7 of these children due to recording error caused by participant non-compliance, inattentiveness, and/or inability to track the eyes. N = 42 children, ages 5 to 16 years (mean age = 8.95 ± 2.59; n = 21 males) participated in this study. We used a broad recruitment strategy in order to obtain a wide range of autism traits. This included identifying participants based on patient referral to our neurodevelopmental pediatric clinic in Lewisburg, Pennsylvania, as well as from health system wide advertisement and the surrounding community. Participants recruited from our clinic were obtained via enrollment in our clinic’s research protocol, which enables access to relevant electronic health record variables as well as recontact of patients for additional research. Our clinic treats children with a very wide range of functioning, including children who would be unable to complete an IQ test. Therefore, we initially screened health records to identify potential participants with an estimated IQ of ~60 or higher and/or the absence of any description of the child being “non-verbal” (i.e. not being able to provide simple responses, use at least two word phrases, understand simple commands). Potential participants were then contacted to complete the screening questionnaire. The brief phone-screening procedure gathered more detailed information regarding the child’s verbal ability, the ability to sit in a chair for approximately 2–3 minutes at a time, and any visual or visual spatial impairments. Exclusionary criteria based upon results from the screening procedure for all interested individuals included: (a) known sensory deficits (e.g. blind or deaf); and/or (b) documented and/or parent reported concerns of visual spatial disorder/impairment or delayed visual maturation. All of the above are due to the fact that the current work was embedded within a larger eyetracking battery that included other simplified tasks that required a verbal response (i.e. labeling a shape). Once screened, no child was excluded from research participation. On the day of research testing, all participants completed a cognitive assessment to document IQ. If an IQ test was ascertained as part of their clinic appointment that day (n = 2), we used the clinically ascertained IQ score. All participants had normal or corrected-to-normal vision. All participants assented to protocols approved by the institutional review board (IRB) at the authors’ home institution. Twelve of our participants had a clinical diagnosis of autism or ASD (n = 4 participants had a comorbid diagnosis of at least one of the following: attention deficit/hyperactivity disorder (ADHD), learning disorder, language disorder, and mild to moderate intellectual disability). One participant had a diagnosis of fetal alcohol syndrome with comorbid ADHD and oppositional defiance disorder (ODD). Demographic information can be found in Table 3.

**Table 3:** Means (SDs) of demographic and behavioral data.

	Males (n = 21)	Females (n = 21)	Total Sample (n = 42)	^∞^t (*p*)
Age	8.48 (2.36)	9.43 (2.78)	8.95 (2.59)	1.19 (0.239)
			Min: 5	
			Max: 16	
FSIQ	108.26 (15.52)	100.38 (16.26)	104.13 (16.21)	−1.56 (0.126)
			Min: 55	
			Max: 137	
SRS-2				
Total	57.67 (13.80)	56.90 (14.14)	57.29 (13.81)	−0.18 (0.861)
			Min: 39	
			Max: 91	
SCI	57.24 (13.41)	57.00 (14.06)	57.12 (13.57)	−0.06 (0.956)
Social Awareness	58.57 (11.51)	57.95 (12.33)	58.26 (11.78)	−0.17 (0.867)
Social Cognition	55.86 (12.71)	55.52 (12.99)	55.69 (12.70)	−0.08 (0.933)
Social Communication	57.29 (13.07)	56.71 (13.96)	57.00 (13.36)	−0.14 (0.892)
Social Motivation	55.43 (13.06)	55.67 (14.68)	55.55 (13.72)	0.06 (0.956)
RBRI	57.38 (14.70)	54.81 (12.41)	56.09 (13.50)	−0.61 (0.544)

^∞^T-scores indicate results from group comparisons (male and female) across Full Scale IQ (FSIQ) and SRS-2 scores. There were no significant differences between males and females (p < 0.05) in age, FSIQ, and SRS-2 scores.

Parents of participants completed the Social Responsiveness Scale-2^nd^ Edition (SRS-2)^38, 39^, a parent-report measure which assesses the presence and severity of symptoms of social impairment associated with autism. SRS-2 Total T-scores can be used to assess symptom severity based upon a provided range: (1) ≤59 T-score: within normal limits/not clinically significant; (2) 60–65 T-score: mild range; (3) 66–75 T-score: moderate range; (4) ≥76 T-score: severe range. In addition to a total score reflecting overall impairments and social communication impairments (SCI), the SRS-2 generates scores across five subscales (Social Cognition, Social Motivation, Social Awareness, Social Communication, and Restricted Interests and Repetitive Behaviors). Average T-scores for all participants were: SRS Total = 57.29 ± 13.81; SCI = 57.12 ± 13.57; Social Awareness = 58.26 ± 11.78, Social Cognition = 55.69 ± 12.70, Social Communication = 57.00 ± 13.36; Social Motivation = 55.55 ± 13.72, Restricted Interests and Repetitive Behaviors = 56.09 ± 13.50. SRS scores for the subset of N = 12 participants with ASD can be found in Table 4.

**Table 4:** SRS-2 T-Scores for ASD subsample.

	ASD subsample (N = 12)	
	**Mean(SDs)**	**Range**
SRS-2		
Total	72.91 (11.65)	Max: 91
		Min: 55
SCI	72.00 (11.46)	Max: 91
		Min: 54
Social Awareness	71.67 (10.54)	Max: 90
		Min: 55
Social Cognition	67.33 (11.60)	Max: 86
		Min: 45
Social Communication	69.33 (11.59)	Max: 90
		Min: 48
Social Motivation	71.58 (11.33)	Max: 90
		Min: 54
RBRI	710.25 (12.90)	Max: 90
		Min: 42

### Task and Procedure

Participants completed a passive viewing eye tracking task during which alternating dark (black screen) and light (white screen) stimuli were displayed. The task began with the presentation of a gray screen (hue: 171.7; saturation: 0.79; brightness: 39.11) for 10 seconds. Baseline pupil diameter was extracted during this time. Following the presentation of the gray screen, the passive viewing task began. See Fig. 1 for task schematic. Stimuli included a black screen with a gray square and a white screen with a gray square. Each stimulus was displayed for 5 seconds before switching to the alternate stimulus display. Participants were instructed to keep their “eyes focused on the center of the square” on the screen during testing. Participants completed 24 total trials (12 black screen trials, and 12 white screen trials). The stimulus presentation order (i.e. whether a participant began the task with a white stimulus display or black stimulus display) was counterbalanced across participants.

Stimuli were presented on a 21.5-inch display monitor via Tobii Studio that allowed for concurrent eye gaze monitoring and pupillometry data acquisition. Testing was done in a quiet, darkened room separate from an experimenter control room via a wall with a two-way mirror. Across each testing session, one experimenter was positioned within the control room while a second experimenter was in the testing room with the participant. The experimenter in the testing room explained all instructions to the participant and ensured the participant remained focused and on task throughout the session. All participants were positioned at a distance of 55–65 cm from the display screen and completed the standard Tobii Studio, 5 point calibration procedure prior to the start of testing. A Symetrix Solus 4 audio mixer allowed for communication between the participant and experimenters between the two rooms. Gaze behavior and eye position was monitored throughout the testing session on a separate monitor in the experimental control room to ensure continuous data collection.

### Eye tracking Data and Analysis

Data was exported from Tobii Studio and subsequent analysis proceeded using adapted MATLAB scripts^53^ and SPSS. In the event of missing data from one pupil, missing values were replaced with the recorded value for the other eye. In the event that missing values existed for both eyes, a linear interpolation was used. Pupil response across each trial and each condition was averaged and smoothed using a low pass (15 Hz) filter. Average baseline pupil diameter was extracted from the 10 second gray screen presented at the start of the task.

Differences in sustained pupil response across dark and light conditions were measured as changes in pupil diameter relative to the average baseline pupil diameter measured at the start of the task. Measures of reflexive changes in pupil response transitioning from one condition to the other were then averaged across each condition. See Fig. 1 (a) dark condition and (b) light condition. Two components of the pupil adaptation response were of interest in the current analysis: (1) the *amplitude* of dilation (*A*_D_) or constriction (*A*_C_) across each condition as well as (2) the *latency* to reach maximum dilation (t_DL_) or constriction (t_CL_) across each condition (See Fig. 2 for visual depiction of these metrics). In order to confirm that task parameters produced distinct differences in pupil constriction and dilation, these measures including average baseline pupil diameter were then entered in a 2 (condition) x 2 (gender) x 4 (pupil metrics) multivariate analysis of variance (MANOVA). Our main analyses focused on (1) identifying which pupil metrics significantly predicted autism features in our pediatric cohort and (2) investigating the relationship between individual differences in pupil metrics and autism features as measured by the SRS-2. Our pupil metrics as well as age and FSIQ were entered into a stepwise linear regression to identify which variables significantly predicted SRS Total T-score. We followed up this stepwise regression with a partial correlation, controlling for age and FSIQ, between pupil metrics and SRS Total and subscale T-scores.

### Ethics approval and consent to participate

Parents of participants provided written informed consent, and the study was approved by the Geisinger Health System Ethical Review Board in Danville, Pennsylvania. The study was conducted in accordance with the standards specified in the 1964 Declaration of Helsinki and its later amendments or comparable ethical standards.

## Electronic supplementary material

Supplementary Information
