# Supplementary material for: Pupil adaptation corresponds to quantitative measures of autism traits in children
Source: Sci Rep. 2017 Jul 25;7:6476. doi: 10.1038/s41598-017-06829-1 (PMC5526922; doi:10.1038/s41598-017-06829-1)
Supplement: Supplementary file 1 — Supplementary Information [file 41598_2017_6829_MOESM1_ESM.pdf]

SUPPLEMENT: Pupil adaptation corresponds to quantitative measures of autism traits in children

*Antoinette Sabatino DiCriscio[A] & Vanessa Troiani[A]

A.) Geisinger Health System; Geisinger Autism and Developmental Medicine Institute (ADMI); Lewisburg, PA

*Corresponding Author:

Antoinette Sabatino DiCriscio, PhD; asdicriscio@geisinger.edu

Geisinger Health System; Geisinger Autism and Developmental Medicine Institute

Lewisburg, PA 17837

		R value<br>(*p* value)											
∞Control Variables		Baseline	t_{CL}	t_{DL}	A_C	A_D	SRS Total	SRS SCI	RBRI	Soc Awr	Soc Cog	Soc Com	Soc Mot
∞Age & FSIQ	Baseline	1.00<br>-											
	t_{CL}	-0.29<br>(0.08)	1.00<br>-										
	t_{DL}	-0.49**<br>(0.002)	0.16<br>(0.32)	1.00<br>-									
	A_C	-0.42**<br>(0.008)	0.21<br>(0.20)	0.65**<br>(<0.001)	1.00<br>-								
	A_D	-0.11<br>(0.50)	0.45**<br>(0.004)	0.34*<br>(0.04)	0.64**<br>(<0.001)	1.00<br>-							
	SRS Total	0.16<br>(0.34)	-0.35*<br>(0.03)	-0.28<br>(0.09)	-0.38*<br>(0.02)	-0.42**<br>(0.008)	1.00<br>-						
	SRS SCI	0.15<br>(0.36)	-0.36*<br>(0.03)	-0.28<br>(0.09)	-0.41**<br>(0.01)	-0.46**<br>(0.003)	0.99**<br>(<0.001)	1.00<br>-					
	RBRI	0.21<br>(0.21)	-0.28<br>(0.09)	-0.26<br>(0.11)	-0.27<br>(0.09)	-0.26<br>(0.12)	0.90**<br>(<0.001)	0.85**<br>(<0.001)	1.00<br>-				
	Soc Awr	-0.006<br>(0.97)	-0.24<br>(0.14)	-0.14<br>(0.39)	-0.27<br>(0.11)	-0.34*<br>(0.04)	0.82**<br>(<0.001)	0.84**<br>(<0.001)	0.67**<br>(<0.001)	1.00<br>-			
	Soc Cog	0.07<br>(0.69)	-0.34*<br>(0.04)	-0.23<br>(0.17)	-0.38*<br>(0.02)	-0.51**<br>(<0.001)	0.90**<br>(<0.001)	0.92**<br>(<0.001)	0.73**<br>(<0.001)	0.73**<br>(<0.001)	1.00<br>-		
	Soc Com	0.21<br>(0.23)	-0.38*<br>(0.02)	-0.27<br>(0.09)	-0.39**<br>(0.01)	-0.47**<br>(0.003)	0.97**<br>(<0.001)	0.98**<br>(<0.001)	0.83**<br>(<0.001)	0.80**<br>(<0.001)	0.88**<br>(<0.001)	1.00<br>-	
	Soc Mot	0.21<br>(0.22)	-0.27<br>(0.10)	-0.33*<br>(0.04)	-0.40*<br>(0.01)	-0.30<br>(0.07)	0.89**<br>(<0.001)	0.87**<br>(<0.001)	0.84**<br>(<0.001)	0.63**<br>(<0.001)	0.71**<br>(<0.001)	0.83**<br>(<0.001)	1.00<br>-

Note: * indicates correlation is significant at the 0.05 level (two-tailed); ** indicates correlation is significant at the 0.01 level (two-tailed)

Abbreviations: Baseline = baseline pupil diameter; t_{CL} = latency to constrict; t_{DL}= latency to dilate; A_C= amplitude of constriction; A_D= amplitude of dilation; SRS SCI= Social Communication Impairment; RBRI= SRS Repetitive Behaviors and Restricted Interests; Soc Awr= Social Awareness; Soc Cog= Social Cognition; Soc Com= Social Communication; Soc Mot= Social Motivation
